# Inhibitory Effects of the Fungal Pigment Rubiginosin C on Hyphal and Biofilm Formation in *Candida albicans* and *Candida auris*

**DOI:** 10.3390/jof9070726

**Published:** 2023-07-05

**Authors:** Haoxuan Zeng, Marc Stadler, Wolf-Rainer Abraham, Mathias Müsken, Hedda Schrey

**Affiliations:** 1 Department of Microbial Drugs, Helmholtz Centre for Infection Research GmbH and German Centre for Infection Research (DZIF), Partner Site Hannover/Braunschweig, Inhoffenstrasse 7, 38124 Braunschweig, Germany; haoxuan.zeng@helmholtz-hzi.de (H.Z.); marc.stadler@helmholtz-hzi.de (M.S.); wrabraham253@gmail.com (W.-R.A.); 2 Institute of Microbiology, Technische Universität Braunschweig, Spielmannstraße 7, 38106 Braunschweig, Germany; 3 Central Facility for Microscopy, Helmholtz Centre for Infection Research GmbH, Inhoffenstrasse 7, 38124 Braunschweig, Germany

**Keywords:** *Candida auris*, *Candida albicans*, biofilms, pseudohyphae, hyphae, extracellular vesicles, drug resistance, virulence

## Abstract

The two fungal human pathogens, *Candida auris* and *Candida albicans*, possess a variety of virulence mechanisms. Among them are the formation of biofilms to protect yeast against harsh conditions through the development of (pseudo)hyphae whilst also facilitating the invasion of host tissues. In recent years, increased rates of antifungal resistance have been associated with *C. albicans* and *C. auris*, posing a significant challenge for the effective treatment of fungal infections. In the course of our ongoing search for novel anti-infectives, six selected azaphilones were tested for their cytotoxicity and antimicrobial effects as well as for their inhibitory activity against biofilm and hyphal formation. This study revealed that rubiginosin C, derived from stromata of the ascomycete *Hypoxylon rubiginosum*, effectively inhibited the formation of biofilms, pseudohyphae, and hyphae in both *C. auris* and *C. albicans* without lethal effects. Crystal violet staining assays were utilized to assess the inhibition of biofilm formation, while complementary microscopic techniques, such as confocal laser scanning microscopy, scanning electron microscopy, and optical microscopy, were used to investigate the underlying mechanisms. Rubiginosin C is one of the few substances known to effectively target both biofilm formation and the yeast-to-hyphae transition of *C. albicans* and *C. auris* within a concentration range not affecting host cells, making it a promising candidate for therapeutic intervention in the future.

## 1. Introduction

Antimicrobial resistance has been a significant and ongoing challenge to public health and manifests itself when pathogenic species become able to eliminate or withstand antibiotic treatments [1,2]. Among the various factors contributing to antimicrobial recalcitrance in bacterial and fungal diseases, biofilm populations play a critical role. According to the National Institute of Health, biofilm formation is linked to 65% of microbial and 80% of chronic infections [3].

*Candida* species are typically commensal yeasts that inhabit human skin and mucosal surfaces. However, they can also cause both superficial and life-threatening systemic infections in the human body, such as oral or vaginal candidiasis, as well as nosocomial bloodstream infections [4,5]. The pathogenicity of *Candida* species is driven by the expression of specific virulence factors, such as biofilm formation, yeast-to-hyphae transition, or the secretion of proteolytic and lipolytic enzymes, which are associated with high mortality rates of infections in hospital settings [6,7].

The process of biofilm formation in *Candida* involves four stages: (i) attachment of yeast cells to a solid surface (within 90 min); (ii) formation of hyphae or pseudohyphae and involvement in biofilm formation; (iii) elongation of hyphae and growth of other polymorphic cells to form an extracellular matrix; (iv) release of new yeast cells [8,9,10,11]. The formation of biofilms not only facilitates the attachment on polymeric surfaces, for example, on medical devices introduced into the human body, but also promotes drug resistance [8]. The underlying resistance mechanisms might be multifactorial, including, e.g., extracellular matrix polysaccharides and efflux pumps [9]. While polysaccharides can impede the activity of antifungal agents by preventing them from reaching their target sites, an increased efflux pump activity can transport antibiotics from inside the cell to the exterior environment [9].

Among all *Candida* species, *C. albicans* is considered to be the most commonly found fungus, causing approximately 50–90% of candidiasis infections [7]. Compared to other *Candida* species, *C. albicans* can produce denser biofilms [11]. Another emerging nosocomial pathogen is *C. auris*, which was first described in 2009 in an external ear canal in a patient from Japan [12]. After its initial identification, numerous outbreaks of invasive infections have been reported in hospitals across several countries and it is rapidly spreading worldwide [13,14,15]. *Candida auris* is resistant to multiple drugs, especially azoles (e.g., fluconazole), polyenes (e.g., amphotericin B), and echinocandins (e.g., caspofungin) [16], and can survive in high-salt and high-temperature environments [17]. It forms biofilms that colonize the skin and can persist on medical device surfaces for up to 14 days [18], leading to infections such as bloodstream infections, urinary tract infections, and invasive candidiasis with high mortality rates [19]. Due to its resistance and ability to form biofilms, it is difficult to eradicate [9]. In general, the biofilms of *C. auris* are thinner than that of *C. albicans*, although the ability to form biofilms varies among *C. auris* isolates [20,21]. Furthermore, several studies have indicated that the pathogenicity of *C. auris* is comparable to, or even more virulent than, that of *C. albicans* [6,22].

Fungi are prolific producers of structural complex secondary metabolites with various biological activities. Over the last century, they have provided several lead structures and pharmacophores that benefit humans. In recent years, novel azaphilones have been identified that exhibit a wide range of biological activities, including antimicrobial, antifungal, antiviral, antibiofilm, antitumor, cytotoxic, and anti-inflammatory activities [23,24,25]. Ascomycetes of the family Hypoxylaceae (Xylariales) are by far the most versatile producers of azaphilones [26]. Due to their chemotaxonomic significance, the pigments have already been successfully consulted to discriminate within the genera and species [27,28,29]. The Hypoxylaceae currently comprises 15 genera (www.mycobank.org; accessed on 3 May 2023), such as *Hypoxylon*, *Daldinia*, or *Jackrogersella*, inhabiting a multitude of habitats, e.g., as endophytes or insect-associates, colonizing lichens, or stromata on decaying wood [26]. It was recently shown that highly complex azaphilones were present in archaeological specimens of *Hypoxylon fragiforme*, estimated to be over 1000 years old [30]. Here, we focused on rubiginosin- and rutilin-type azaphilones, obtained from the stromata of the ascomycetes *Hypoxylon rubiginosum* and *Hypoxylon texense*, which have the ability to produce the pigments around the perithecia [30,31,32]. Weak to moderate antimicrobial activities of some rubiginosin- and rutilin-type azaphilones against *Bacillus subtilis* and *Staphylococcus aureus* were previously reported [28].

**Figure 1:**
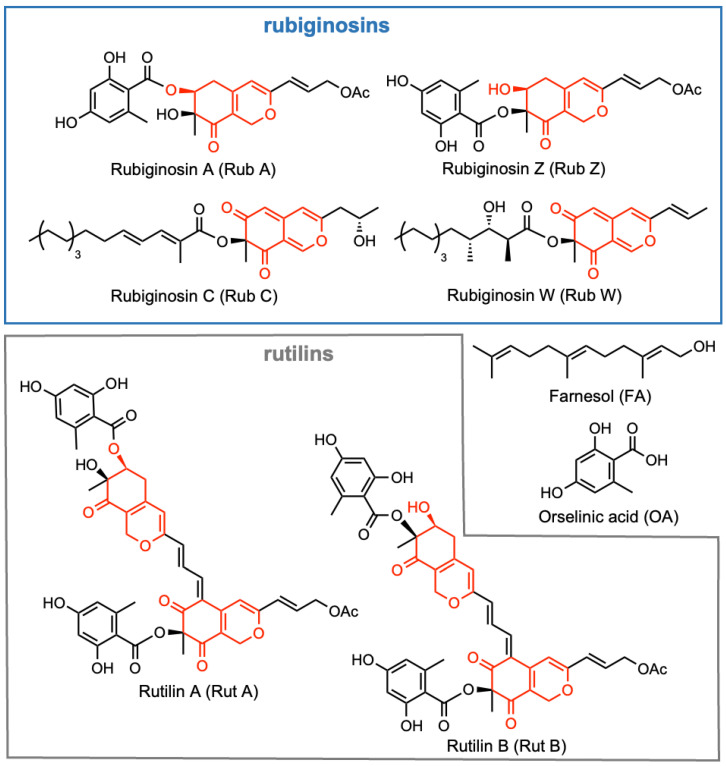
Chemical structures of selected rubiginosin- and rutilin-type azaphilones together with farnesol (FA) and orsellinic acid (OA); azaphilone core shown in red.

In the course of our ongoing search for novel anti-infectives, we conducted a study to investigate the potential of the selected azaphilones (Figure 1) for their inhibitory efficacy against (pseudo)hyphae development and their effects on biofilms. Our investigation focused on their effectiveness against the two invasive fungal pathogens *C. albicans* and *C. auris*. Although all pigments share the same pyronoquinone core, they differ in the type of substituents. Thus, rubiginosin C (Rub C) and rubiginosin W (Rub W) are substituted by a linear polyketide moiety of different lengths and substitution patterns. In contrast, the monomeric rubiginosin A (Rub A) and rubiginosin Z (Rub Z) carry an orsellinic acid (OA) unit, while rutilin A (Rut A) and rutilin B (Rut B) are their dimeric congeners. 

## 2. Materials and Methods

### 2.1. Isolation of Selected Azaphilones and Preparation of Pathogenic Strains

Isolation of all azaphilones was achieved in previous work by Becker et al. [28], and aliquots were used herein (Supplementary Material for detailed information).

Strains *C. auris* [DSM 21092] and *C. albicans* [DSM 1665, DSM 11225] were obtained from the German Collection of Microorganisms and Cell Cultures GmbH (DSMZ, Braunschweig, Germany) as a freeze-dried sample. The organisms were cultured in Yeast Extract Peptone Dextrose (YPD) medium (30 °C, 120 rpm, 2 d). The 1 mL aliquots with 20% glycerol were stored at −20 °C for short-term use, and −80 °C for long-term use. Strain *C. albicans* CAI-4 *HWP*1-*lacZ* [ZK3379] [33] was provided by Prof. Ursula Bilitewski (Helmholtz Centre for Infection Research GmbH, Braunschweig, Germany) in 1 mL aliquots, stored in 20% glycerol at −20 °C. 

### 2.2. Determination of Minimum Inhibitory Concentration (MIC) and Cytotoxicity 

The MICs of rubiginosin- and rutilin-type azaphilones—in detail, Rub A, C, W, Z, and Rut A and B—were determined as described previously [34] using the selected pathogens *C. albicans* [DSM 11225, DSM 1665], *C. albicans* CAI-4 *HWP1*-*lacZ* [ZK3379] and *C. auris* [DSM 21092] (see Table S1 for detailed information). All compounds were tested within the concentration range of 250 µg/mL–2 µg/mL for *C. albicans* [DSM 11225, DSM 1665], *C. albicans* CAI-4 *HWP1-lacZ* [ZK3379], *C. auris* [DSM 21092].

Cytotoxic effects were evaluated on human endocervical adenocarcinoma KB-3-1 [ACC 158] cells and mouse fibroblasts L929 [ACC 2] upon treatment with Rub C, W, and Rut A and B within the concentration range of 37 μg/mL–0.63 ng/mL. The half-maximum inhibitory concentrations (IC_50_) were determined by standard MTT assays as reported previously [32] (see the Supplementary Material for detailed information). The IC_50_ values of Rub A and Z have currently been reported [28].

### 2.3. Antibiofilm Assay with Crystal Violet

#### 2.3.1. Biofilm Formation Assay of *C. auris* and *C. albicans*

The fungal pathogens *C. auris* [DSM 21095] or *C. albicans* [DSM 11225] were cultured from stock in 25 mL YPD medium in a 250 mL flask (30 °C, 100 rpm, 18 h). The turbidity of the broth was measured at 280 nm using a spectrophotometer (Nanodrop 2000c, Thermo Fisher Scientific, Waltham, MA, USA) and diluted to match the turbidity of a 0.5 McFarland standard in RPMI 1640 medium (Gibco, New York, NY, USA; Thermo Fisher Scientific), supplemented with 0.165 mM 3-(N-morpholino)propanesulfonic acid (MOPS, Carl Roth, Karlsruhe, Germany) for *C. auris* [35,36], or 0.05 McFarland standard in RPMI 1640 medium in the case of *C. albicans* [37,38,39]. Subsequently, 150 μL of the fungal dispersion were added into each well of a 96-well microtiter plate (Falcon no. 351172, Thermo Fisher Scientific) and further incubated (37 °C, 150 rpm, 2 h [*C. auris*] or 90 min [*C. albicans*]). The supernatant was discarded and the plate was rinsed one time by using a PBS buffer. Afterwards, azaphilones (Rub A, C, W, Z, and Rut A and B) were serially diluted in 150 μL in fresh medium to concentrations of 250–2 μg/mL (Rub C for *C. auris*: 250–0.02 μg/mL). Methanol (2.5%) was used as a solvent control and both, nystatin (NYS; Thermo Fisher Scientific) and farnesol (FA; Sigma Aldrich, St. Louis, MO, USA) as positive controls (250–2 μg/mL) for *C. auris* and *C. albicans*, respectively. Plates were further incubated (37 °C, 150 rpm, 24 h). The supernatant was discarded and biofilms were washed with PBS, stained by adding 150 μL of the crystal violet (CV; Sigma Aldrich) solution (0.1%), and incubated (room temperature, 25 min). Afterwards, the plates were washed twice with PBS buffer. A total of 150 μL ethanol (95%) were applied to dissolve the biofilm-bound CV. The absorbance was measured by a plate reader (Synergy 2, BioTek, Santa Clara, CA, USA) at 570 nm for *C. auris* or 610 nm for *C. albicans*. Error bars indicate SD with duplicates in two biological repeats.

#### 2.3.2. Assay to Determine Rub C Effects on *C. auris* Biofilms of Various Ages

The turbidity of *C. auris* [DSM 21092] dispersion was measured at 280 nm and diluted to the turbidity of a 0.5 McFarland standard. *C. auris* was cultured in RPMI 1640 medium supplemented with 0.165 mM MOPS (37 °C, 150 rpm, 2 h, 12 h, 24 h) in 96-well non-tissue microtiter plates (Falcon no. 351172, Thermo Fisher Scientific) [40]. After incubation, *C. auris* biofilms of various ages (2 h, 12 h, 24 h) were washed once by PBS buffer and treated (37 °C, 150 rpm, 24 h) with serial diluted Rub C (250–0.02 μg/mL) in fresh RPMI 1640 medium supplemented with 0.165 mM MOPS. Samples of each time point were further processed and evaluated by a microtiter plate reader, as described above.

### 2.4. Observations of Biofilm by Confocal Laser Scanning Microscopy (CLSM) 

A culture of *C. auris* [DSM 21092] was adjusted to the turbidity of a 0.5 McFarland standard and cultured (37 °C, 2 h) in RPMI 1640 medium supplemented with 0.165 mM MOPS to allow for the attachment of fungal cells to μClear microtiter plates (Greiner Bio-One, Kremsmünster, Austria) [41]. Afterwards, wells were gently rinsed with PBS buffer and incubated with 150 μL of fresh medium containing Rub C in the concentrations 250 and 15.6 μg/mL, respectively. Medium with 2.5% methanol was used as a solvent control. The plates were covered with an air-permeable breath seal cover foil (Greiner Bio-One) and further incubated (37 °C, 24 h). Afterwards, the supernatant with planktonic cells was slowly removed with a multi-channel pipette. Biofilms were gently washed once with PBS buffer and stained with the fluorescent dyes FUN-1 (Invitrogen, Waltham, MA, USA; Thermo Fisher Scientific) and Calcofluor White M2R (Invitrogen, Thermo Fisher Scientific) by incubating (37 °C, 30 min) the wells with 150 μL PBS containing 10 μM FUN-1 and 25 μM M2R in the dark [42]. Plasma membrane integrity and metabolic function of fungi are required to convert the yellow-green-fluorescent intracellular staining of FUN 1 into the red-orange-colored intravascular structures. This contrasts with the yellow-green fluorescence of dead cells where FUN-1 remains in the cytosol. Calcofluor White M2R labels cell-wall chitin and beta glucoside bonds appearing as green, fluorescent signals regardless of the metabolic state of the cell. Stained biofilms were observed using an inverted, confocal laser scanning microscope SP8 (Leica Microsystems, Wetzlar, Germany) and acquired with the software LAS X and the following settings: Z-step size is 2 μm, green (excitation = 488 nm and emission = 530 nm) and red (excitation = 488 nm and emission = 620 nm) fluorescence signal. Image analysis was performed with the software Image J 1.53 k (National Institute of Health, Bethesda, MD, USA) for quantification and Imaris 9.31 (Oxford, UK) for visualization.

### 2.5. Colony Forming Units (CFU) and the Growth Curve of Candida

#### 2.5.1. CFU of *C. auris*

The preculture of *C. auris* [DSM 21092] was prepared and adjusted to the turbidity as previously described in Section 2.4. After 2 h of incubation, Rub C was added to each well to the final concentrations of 250 µg/mL and 15.6 µg/mL. CFU was tested after 12 and 24 h, respectively. Cells were resuspended in the well 50 times. We prepared a dilution series in 1 to 10 steps (20 µL in 200 µL) down to a final dilution level of 10^−6^ and platted 100 µL of this last dilution on YPD agar plates using small glass beads (5 to 10) to homogeneously spread the liquid. Individual yeast colonies on the non-hyphal promoting agar plates were counted after incubation at 30 °C for 2 days [43]. Afterwards, CFUs were calculated considering the dilution factors. Error bars indicate SD with duplicates in two biological repeats. 

#### 2.5.2. The Growth Curve of *Candida*

The preculture of *C. auris* [DSM 21095] and *C. albicans* [DSM 11225] were adjusted to a 0.1 McFarland standard, cultured (37 °C, 150 rpm) in RPMI 1640 medium supplemented with 0.165 mM MOPS and RPMI 1640 medium, respectively, and added together with Rub C to a 96-well microtiter plate (Falcon no. 351172, Thermo Fisher Scientific) to result in concentrations of 250 µg/mL and 62.5 µg/mL [44]. The absorbance was measured at 630 nm after 2 h, 5 h, 8 h, 14 h, and 20 h. Methanol (2.5%) was used as a solvent control. Error bars indicate SD with duplicates in two biological repeats. 

### 2.6. Observations of Candida Cells by Optical Microscopy

#### 2.6.1. Visualization of *C. auris* and *C. albicans* Cells

Planktonic cells of *C. auris* [DSM 21092] or *C. albicans* [DSM 11225] were incubated with different concentrations of Rub C in 96-well non-tissue microtiter plates (37 °C, 150 rpm, 24 h; *C. auris*: with 250, 15.6 μg/mL of Rub C in 150 μL RPMI 1640 medium with 0.165 mM MOPS; *C. albicans*: with 250, 62.5 μg/mL of Rub C in 150 μL RPMI 1640 medium with 50 nM glucose (Sigma Aldrich) and 50 mM 4-(2-hydroxyethyl)-1-piperazineethanesulfonic acid (HEPES, Sigma Aldrich)) [44]. After 24 h of incubation, 30 μL of 25% paraformaldehyde (PFA, Thermo Fisher Scientific) were added into each well. Planktonic cells were fixed at room temperature for 30 min and the supernatant of each well was taken out and collected in a 1.5 mL Eppendorf tube (Lot. I182541I, Eppendorf, Hamburg, Germany) following centrifugation with 12,000 rpm for 12 min at room temperature. The supernatant was removed and the pellet was resuspended in 20 μL PBS buffer. Afterwards, 15 μL resuspended cells were loaded on microscope slides (LOT 7691777, Thermo Fisher Scientific) and covered with cover glass (24 × 50 mm Menzel-Gläser, Braunschweig, Germany). Samples were monitored using an Axio Imager A2 light microscope equipped with a 63×/1.25 oil objective (Zeiss, Jena, Germany) and the Zen blue 3.0 software. 

#### 2.6.2. Assay to Determine the Impact of Rub C on Hyphae

*C. albicans* [DSM 11225] was cultured (37 °C, 150 rpm, 24 h and 48 h) in 96-well non-tissue microtiter plates in RPMI 1640 medium supplemented with 50 nM glucose and 50 mM HEPES to form hyphae. Afterwards, 24 h- and 48 h-old hyphae were treated with Rub C (62.5 μg/mL) and cultured (37 °C, 150 rpm, 2 h, 5 h, 10 h, and 18 h) in fresh RPMI 1640 medium supplemented with 50 nM glucose and 50 mM HEPES for various periods of time [44]. Each sample was fixed as described in Section 2.6.1 and monitored using an Axio Imager A2 light microscope equipped with a 63×/1.25 oil objective (Zeiss) and the Zen blue 3.0 software. 

### 2.7. Observations of Candida Cells by Scanning Electron Microscopy (SEM)

*C. auris* [DSM 21092] was incubated (37 °C, 24 h) with Rub C at the concentration of 250 and 15.6 μg/mL in 96-well non-tissue microtiter plates in RPMI 1640 medium supplemented with 0.165 mM MOPS. Cultures were fixed with 5% formaldehyde and 2% glutaraldehyde (final concentrations) and washed twice in TE buffer (20 mM TRIS with 1 mM EDTA, pH 6.9). A 50 µL aliquot was added to round poly-l-lysine pretreated coverslips and incubated (room temperature, 10 min). Further processing was carried out as previously described with slight modifications [45]. In brief, cells were fixed for 10 min on the coverslip with TE buffer including 1% glutaraldehyde (final concentration). The coverslips were washed twice in TE buffer and dehydrated in 10 min steps on ice with a graded series of acetone (10%, 30%, 50%, 70%, and 90%), followed by two steps in 100% acetone at room temperature. The coverslips were mounted onto aluminum stubs with carbon adhesive discs; they were critical-point-dried with the automated CPD300 (Leica Microsystems) and gold-palladium-sputter-coated (55 s at 45 mA) with a SCD500 (Bal-Tec, Balzers, Liechtenstein). Images were acquired with a field emission scanning electron microscope Zeiss Merlin (Zeiss, Oberkochen, Germany) using the Everhart Thornley HESE2 detector and the in lens SE detector in a 25:75 ratio with an acceleration voltage of 5 kV.

### 2.8. Screening and Quantification of Hyphal Inhibitory Activities with β-Galactosidase Activity Assay

The strain *C. albicans* CAI-4 *HWP1*-*lacZ* was directly precultured overnight from a cryo-stock in a defined medium (6.7 g/L yeast nitrogen base without amino acids, 9 g/L glucose, 1 g/L maltose) at 30 °C [46]. Cells were washed twice in pre-warmed hyphae-inducing medium SLAD (1.7 g/L yeast nitrogen base without amino acids without ammonium sulfate, 2 g/L glucose, 1 g/L maltose, 6 mg/L ammonium sulfate, buffered to pH 7.3 using 0.165 M MOPS) and resuspended in the same medium. The turbidity of fungal dispersion was set to a 0.1 McFarland standard (280 nm). A total of 50 μL of the mixed suspension was added to each well of a 96-well microtiter plate containing azaphilones (Rub A, C, W, Z, and Rut A and B), serially diluted to concentrations of 100–6.3 μg/mL, and FA was used as a positive control (100–0.8 μg/mL). Afterwards, the 96-well microtiter plate was incubated (37 °C, 150 rpm, 5 h) to induce hyphal growth. 

After, hyphae induction cells were incubated (37 °C, 150 rpm, 60 min) with 100 μL of z-buffer (composed of 60 mM Na_2_HPO_4_, 40 mM NaH_2_PO_4_, 10 mM KCl, 1 mM Mg_2_SO_4_, 1 mM DTT, and 0.2% sodium lauroyl sarcosinate) to lyse the cells. Following this, 50 μL of 4 g/L o-nitrophenyl beta-d-galactopyranoside (ONPG, Sigma Aldrich) supplemented with 0.1 M potassium phosphate buffer (pH 7.0) was added, and the optical density was measured at 414 nm and 550 nm wavelengths using a microtiter plate reader (Synergy 2, BioTek), and was measured again after 120 min incubation at 37 °C to determine the hydrolysis of ONPG due to β-galactosidase activity. The absorption by o-nitrophenol was calculated as follows: (OD_414 nm_ − c × OD_550 nm_)_120 min_ − (OD_414 nm_ – c × OD_550 nm_)_0 min_. c was determined as 1.36 when using microtiter plates as shown in the study of Heintz-Buschart et al. [47].

### 2.9. Statistical Analysis

Differences between samples and the control group were determined by a two-tailed Student’s *t*-test. Statistical significance was defined as *p* < 0.05. Analysis was carried out using GraphPad Prism 9^®^ (GraphPad Software, San Diego, CA, USA) [48].

## 3. Results

### 3.1. Determination of MIC and Cytotoxicity of Azaphilones

In accordance with previous results, no antimicrobial activity was observed for the six selected azaphilones (Rub A, C, W, Z, and Rut A and B) against the four strains of *Candida* (*C. albicans* [DSM 1665, DSM 11225], CAI-4 *HWP1*-*lacZ*, and *C. auris*) at the highest tested concentration of 250 µg/mL (Table 1) [28]. 

Only the dimeric rutilin-type azaphilone derivatives Rut A and Rut B exhibited significant cytotoxic activity with IC_50_ values in a range of 1.1–2.2 μM (0.9–1.8 µg/mL) against murine fibroblasts (L929) and human endocervical adenocarcinoma cells (KB-3-1) (Table 2). Furthermore, moderate cytotoxicity against these two cell lines with IC_50_ values between 3.2 and 5.4 μM (2.6–4.5 µg/mL) has been reported for monomeric OA-carrying Rub A and Rub Z in a previous study by Becker et al. [28].

### 3.2. Inhibitory Effects of Azaphilone Pigments against Candida Biofilm Formation

**Figure 2:**
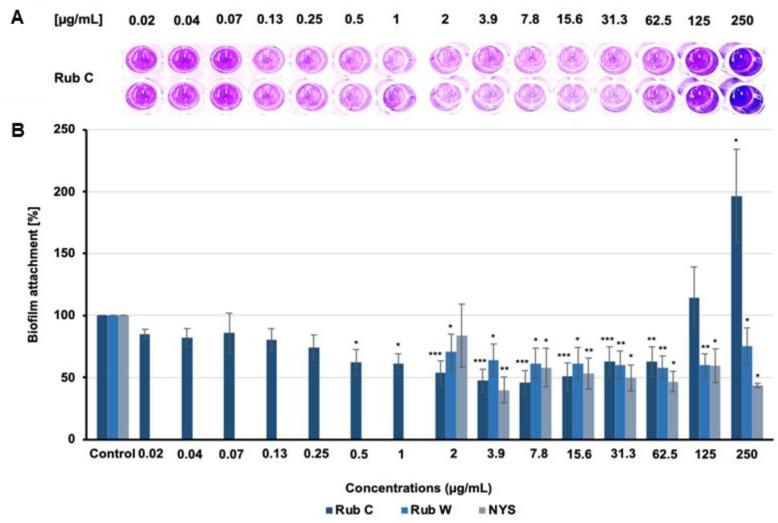
Effect on the biofilm formation of *C. auris* after 24 h treatment with Rub C and Rub W. (**A**) Image of CV-stained wells of a microtiter plate. (**B**) Efficacy of Rub C and Rub W on the formation of *C. auris* biofilms. Nystatin (NYS) was used as positive control, methanol as solvent control. Error bars indicate SD of duplicates in two biological repeats; *p* values: * *p* < 0.05, ** *p* < 0.01, *** *p* < 0.001.

**Figure 3:**
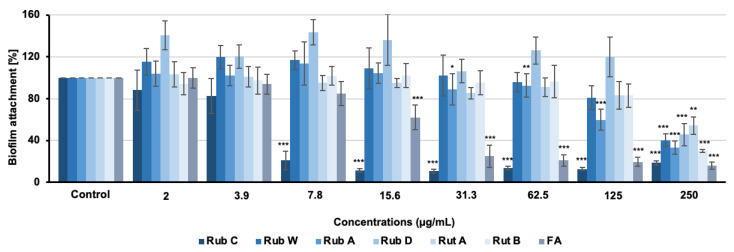
Effect on biofilm formation of *C. albicans* after 24 h treatment with rubiginosin- and rutilin-type azaphilones. FA was used as positive control, methanol as solvent control. Error bars indicate SD of duplicates in two biological repeats; *p* values: * *p* < 0.05, ** *p* < 0.01, *** *p* < 0.001.

In addition to MIC assays, we also assessed the efficacy of the six azaphilone derivatives (Rub A, C, W, Z, and Rut A and B) towards biofilms of several pathogens (*C. albicans*, *C. auris*, *Pseudomonas aeruginosa*, and *S. aureus*) by a CV assay. Rub C, W, and Rut A and B exhibited moderate inhibitory effects against the biofilm formation of *S. aureus,* while none of the tested rubiginosin- or rutilin-type azaphilones was active against *P. aeruginosa* (Table S2). Interestingly, moderate activities against the biofilms of *S. aureus* have also been reported for the known hybridorubrins, which are structurally related to Rub C and W [29]. Furthermore, promising inhibitory effects were observed for Rub A, C and W against the biofilm formation of *C. auris* and *C. albicans* at sub-inhibitory concentrations (Figure 2 and Figure 3).

Rub C and Rub W showed prohibitive activity against the formation of *C. auris* biofilms (Figure 2), whereas monomeric and dimeric OA-carrying azaphilones (Rub A, Z and Rut A and B) did not affect the formation (Table S2). Regarding the effects of Rub C, more biofilm mass was observed above the concentration of 125 µg/mL. At a concentration of 250 µg/mL, the absorption was almost doubled compared to the control. At Rub C concentrations between 0.5 and 62.5 µg/mL, we could detect significant inhibitory effects of ca. 50% against the formation of biofilms. Rub W, which is structurally related to Rub C, was slightly less inhibitory. Interestingly, we could not detect a strong increase in biofilm biomass for Rub W at higher concentrations. 

As shown in Figure 3, all tested pigments inhibited the formation of *C. albicans* biofilms when applied at the highest test concentration of 250 µg/mL. Only two rubiginosins, Rub A and C, exhibited activity below this concentration. While Rub A showed significant effects starting at concentrations above 31.3 µg/mL, Rub C exhibited strong inhibitory effects of ca. 50–80% biofilm reduction at concentrations between 7.8 µg/mL and 250 µg/mL (Figure 3); hence, even exceeding the inhibitory activity of FA. 

### 3.3. Rub C Activity against Different Maturation Stages of C. auris Biofilm 

**Figure 4:**
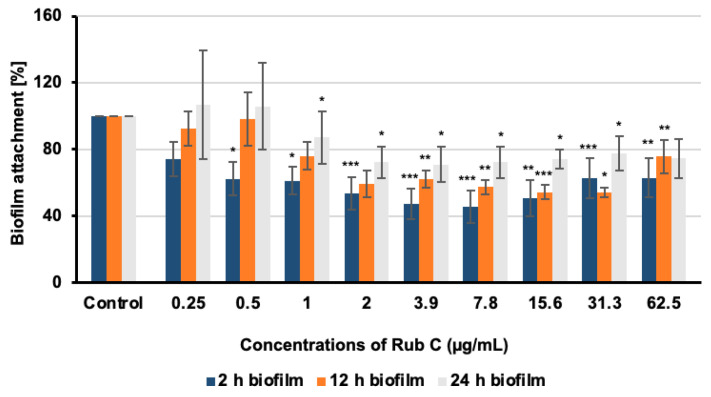
Effect on biofilm formation of pre-grown *C. auris* biofilms of different developmental stages (2 h, 12 h, and 24 h) after 24 h treatment with Rub C. The solvent methanol served as solvent control. Error bars indicate SD of duplicates in two biological repeats; *p* values: * *p* < 0.05, ** *p* < 0.01, *** *p* < 0.001.

To investigate the efficacy of Rub C towards different developmental phases of *C. auris* biofilm formation, the cells were grown for 2 h (attachment phase), 12 h, and 24 h (maturating biofilms), respectively, before medium exchange and treatment with Rub C for 24 h. The drug nystatin was used as the positive control (Table S3). The CV assay revealed that Rub C activity was carried out with a progressing biofilm state (Figure 4).

### 3.4. Visualization of the Effect of Rub C on C. auris Biofilms via CLSM

**Figure 5:**
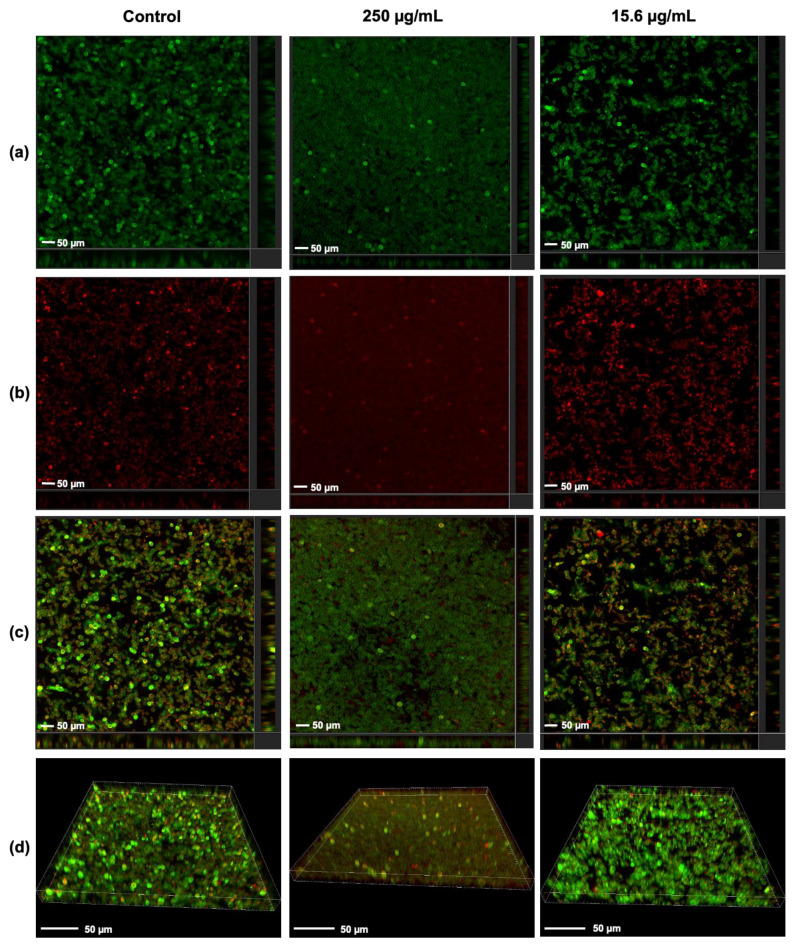
Three-dimensional projections and orthogonal sections of *C. auris* biofilms (24 h after exposure to the Rub C of attached cells [2 h]). The samples were stained with FUN 1 dye and Calcoflour White M2R. Regarding intensive overlapping, green-yellow fluorescence represents dead cells, and intracellular red fluorescence represents metabolically active cells. Treatments were carried out at concentrations of 250 and 15.6 µg/mL at 37 °C in RPMI 1640 (supplemented with 0.165 mM MOPS). (**a**) Calcafluor White M2R. (**b**) FUN-1. (**c**) Multichannel. (**d**) Three-dimensional projections. Exemplary images of 2 independent experiments are shown.

To address the inhibitory effects of Rub C towards *C. auris*, CLSM was used to visualize the three-dimensional structure in the early phase of biofilm development. Analysis of CLSM exhibited morphological variances in the biofilm structures treated with two different concentrations of Rub C (Figure 5). At 250 µg/mL, the morphology of the biofilm was flatter but with densely packed single cells compared to untreated biofilms while less metabolic activity was observed. In contrast, biofilms exposed to 15.6 µg/mL of Rub C appeared more porous and the cells were aggregated and more heterogeneous in size (Figure 5). We also saw differences in the overall biofilm biomass (250 µg/mL > solvent control or 15.6 µg/mL), as shown in Figure S2.

### 3.5. Candida Growth Is Promoted with High Rub C Concentrations

**Figure 6:**
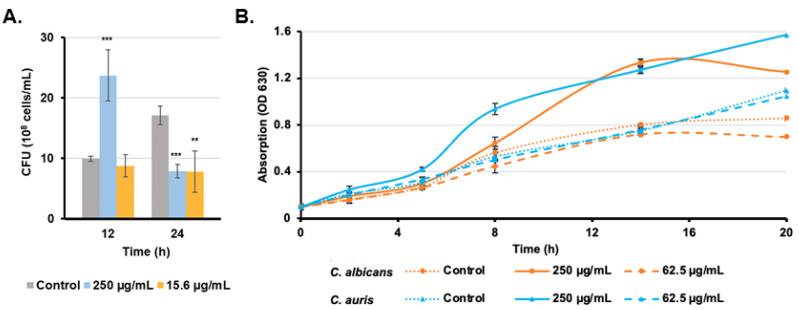
(**A**). Effects of Rub C on the biofilm formation of *C. auris* (CFU). Methanol was used as solvent control. Error bars indicate SD with duplicates in two biological repeats. *p* values: ** *p* < 0.01, *** *p* < 0.001. (**B**). Effects of Rub C on the growth of *C. auris* and *C. albicans* planktonic cells. The absorption (OD 630 nm) was measured after 2 h, 5 h, 8 h, 14 h, 20 h. Methanol was used as the solvent control. Error bars indicate SD with duplicates in two biological repeats.

In addition to CLSM, a CFU assay was conducted from resuspended biofilms in the same experimental setup to determine the effects of Rub C at concentrations of 250 µg/mL and 62.5 µg/mL on biofilms of *C. auris* for two incubation time points: 12 h and 24 h (Figure 6A). After 12 h of treatment, the concentration of 250 µg/mL Rub C resulted in more than double the number of colonies compared to the control, while the sample treated with 15.6 µg/mL Rub C showed similar values to the control. This ratio changed after 24 h: while the control nearly doubled in colony numbers, biofilms exposed to 250 µg/mL Rub C dropped in number and those exposed to 15.6 µg/mL showed no significant change compared to 12 h incubation. These observations are in line with the time-dependent treatment of Rub C within the CV assay, where the strongest effects on the 2 h-old biofilms of *C. auris* were observed after a 24 h treatment (Figure S1). 

In order to further identify the effects of Rub C on the planktonic cells of *C. auris* and *C. albicans*, the optical density was measured in the presence of Rub C at concentrations of 250 µg/mL and 62.5 µg/mL over a period of 20 h. The optical density of *C. auris* and *C. albicans* cultures increased faster and remained up to 1.5-fold higher in the presence of 250 µg/mL Rub C, while both the untreated cultures as well as cultures, which are exposed to 62.5 µg/mL, showed more slowly increasing OD values (Figure 6B). 

### 3.6. Rub C Induces Morphological Changes of C. auris and C. albicans Cells 

**Figure 7:**
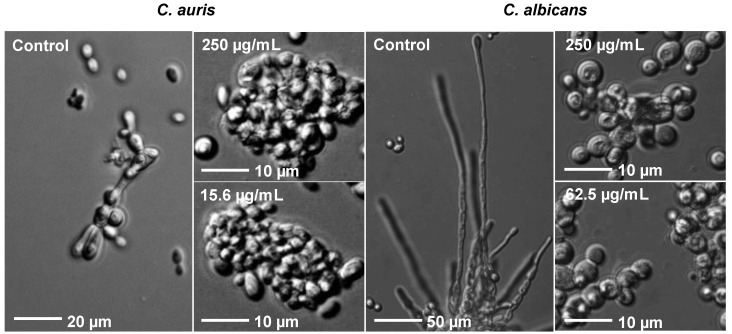
Effects of Rub C on the planktonic cells of *C. auris* and *C. albicans*. Both pathogens were incubated with Rub C in RPMI 1640 (supplemented with 0.165 mM MOPS [*C. auris*] or 50 nM glucose and 50 mM HEPES [*C. albicans*]) at 37 °C for 24 h. All samples were monitored with an optical microscope using a 63× oil objective. Exemplary images of 2 independent experiments are shown.

To further characterize the underlying effects of Rub C on *C. auris* and *C. albicans*, planktonic cells were analyzed via light microscopy after incubation in hyphal−growth−promoting medium RPMI 1640 at 37 °C and in the presence of 15.6 µg/mL and 250 µg/mL Rub C. While the control exhibited the formation of pseudohyphae, no pseudohyphal development was observed for Rub C-treated cells (Figure 7). At both concentrations, mainly aggregated cells could be found.

Furthermore, the dimorphic fungus *C. albicans* also has the ability to grow either as unicellular yeast or in filamentous pseudohyphal or hyphal forms. Comparable to *C. auris*—but significantly more pronounced—were the morphological differences between incubated cells and the control (Figure 7). When Rub C was applied at 250 µg/mL and 62.5 µg/mL, the hyphal growth was inhibited and only aggregated unicellular yeast cells could be observed.

**Figure 8:**
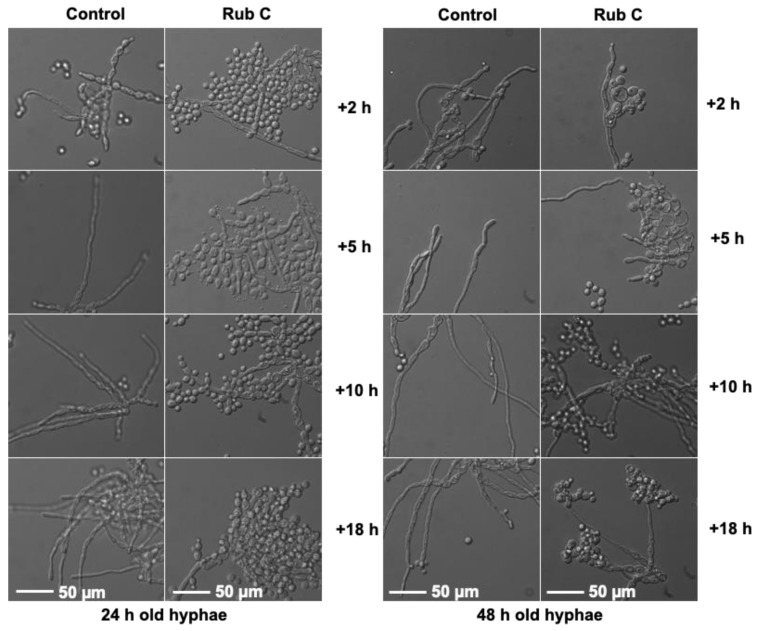
*C. albicans* cultured in hyphal−growth-promoting medium RPMI 1640 (supplemented with 50 nM glucose and 50 mM HEPES) at 37 °C for 24 h or 48 h and treated with 62.5 µg/mL Rub C for 2 h, 5 h, 10 h and 18 h. Conversion of hyphae into yeasts was monitored under a light microscope using a 63× oil objective. Exemplary images of 2 independent experiments are shown.

In order to evaluate whether Rub C could address the morphological switch from the hyphal form of *C. albicans* to its yeast form, preformed hyphae (24 h and 48 h) were treated with 62.5 µg/mL Rub C and monitored after 2 h, 5 h, 10 h, and 18 h. Already after 2 h treatment with Rub C, aggregated yeast cells could be detected in both 24 h- and 48 h-old hyphae. The most pronounced effect was achieved after 18 h incubation, with Rub C being more effective on 24 h-old hyphae than on 48 h-old ones (Figure 8).

**Figure 9:**
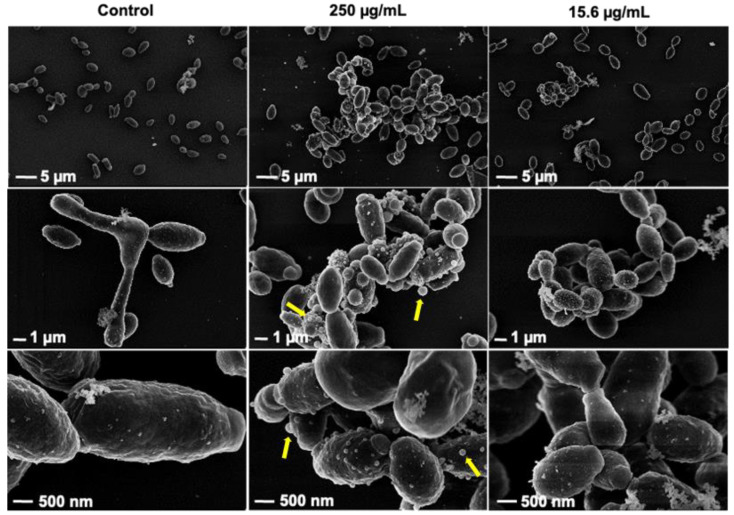
Effects of Rub C on the planktonic cells of *C. auris,* as shown by SEM micrographs. Cells were incubated with Rub C in RPMI 1640 (supplemented with 0.165 mM MOPS) at 37 °C for 24 h and exposed to Rub C with indicated concentrations. Differences with regard to cell morphology and vesicles are observed in the samples treated with 250 µg/mL Rub C (yellow arrows).

In the same setup, we also prepared samples to study *C. auris* cells via SEM. When Rub C was applied at concentrations of 250 µg/mL and 15.6 µg/mL, pseudohyphal development was inhibited. Moreover, we could observe more extracellular vesicles (EVs) in the treated samples, which were especially visible after the treatment with 250 µg/mL Rub C; the size of the surface-bound vesicles was clearly larger (Figure 9).

### 3.7. Quantification of Inhibitory Activities of Azaphilones against C. albicans

**Figure 10:**
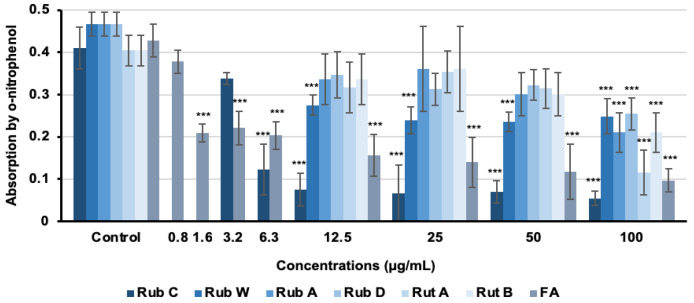
Inhibitory effects of selected rubiginosin- and rutilin-type azaphilones on the hyphal development of *C. albicans*. Production of o-nitrophenol by β-galactosidase expression as a measure of *HWP1* expression after 5 h of incubation. FA was used as the positive control and methanol as solvent control. Error bars indicate SD with duplicates in three biological repeats; *p* values: *** *p* < 0.001.

To evaluate the efficacy of selected rubiginosin- and rutilin-type azaphilones as inhibitors for hyphal induction, hyphae gene expression was quantified using the CAI-4 *HWP1*-*lacZ* reporter strain [44,45]. In this construct, the *HWP1* promotor is combined with the *lacZ* gene. The HWP1 cell wall protein is exclusively produced during hyphae growth, and thus, the *lacZ* gene (under control of the *HWP1* promotor) is only expressed under the same conditions. With the cleavage of the β-galactosidase substrate o-nitrophenyl beta-D-galactopyranoside and the resulting photometrically measurable product o-nitrophenol, hyphae gene expression can be quantified [46,47]. Consequently, higher concentrations of o-nitrophenol corresponded to greater levels of hyphal expression. We determined the β-galactosidase activity after 5 h of treatment of the strain *C. albicans* CAI-4 *HWP1*-*lacZ* with all azaphilones; FA was used as the positive control. The reporter assay exhibited inhibitory effects for all azaphilones when applied at the highest test concentration of 100 µg/mL. Among all pigments, Rub C and Rub W exhibited significant inhibitory effects. Rub C showed the most pronounced inhibitory effects on hyphae gene expression of up to 87% reduction compared to the control when applied at 100 µg/mL (Figure 10). In addition, Rub C was able to effectively inhibit the gene expression of hyphae with 71% efficacy even at the concentration of 6.3 µg/mL (Figure 10).

## 4. Discussion

The discovery of novel therapeutic agents is crucial for combating the growing problem of antibiotic resistance in fungal infections. In this context, azaphilones, a group of fungal secondary metabolites, have been studied extensively for their antimicrobial and cytotoxic properties, gaining increasing attention for their potential as therapeutic agents [24].

In this study, we investigated the activity of selected rubiginosin- and rutilin-type azaphilones against biofilm-related bacterial and fungal pathogens (*S. aureus*, *P. aeruginosa* as well as *C. albicans* [DSM 1665, DSM 11225], CAI-4 *HWP1*-*lacZ*, and *C. auris*) and their cytotoxicity against two cell lines. The fungal pigments did not exhibit any inhibitory activity against all tested strains of *C. albicans* and *C. auris*. Azaphilones bearing OA moiety, such as Rub A and Z, Rut A, and B, demonstrated cytotoxic properties, while azaphilones substituted by an aliphatic moiety instead of the OA motif, like Rub C and W, did not exhibit such effects. Additionally, the dimeric OA-carrying Rut A (1.2 µM; 1.1 µM) and B (2.2 µM; 1.5 µM) demonstrated stronger cytotoxicity against L929 and KB-3-1cell lines than the monomeric OA-carrying azaphilones Rub A (3.2 µM; 5.2 µM) and Z (4.7 µM; 5.2 µM) [28]. These cytotoxic effects might be attributed to the presence of an OA moiety within the molecule [28], although no cytotoxicity could be observed for OA alone [49].

In addition to the antimicrobial properties of the selected azaphilones, we also tested the inhibitory activity against biofilm formation. Among all pigments, Rub C exhibited the most pronounced effects against the biofilm formation of *C. albicans* and *C. auris* without being lethal (MIC > 250 µg/mL) or cytotoxic against the tested cell lines in the tested range (37 μg/mL–0.63 ng/mL). Rub C exhibited promising inhibitory effects against the biofilm formation of *C. auris* (between 2 and 62.5 μg/mL) and *C. albicans* (>7.8 μg/mL) of ca. 50% and ca. 80%, respectively. In contrast, Rub W and A demonstrated weaker activities against the biofilm formation of *C. auris* and *C. albicans*, respectively. At the highest test concentration (250 μg/mL), all azaphilones showed effects towards *C. albicans* biofilm formation. Tentative structure–activity relations let us assume that an increase in lipophilicity affected the inhibitory effects of the selected azaphilones. Thus, azaphilones with a long lipophilic side chain exhibited stronger inhibitory effects against biofilm formation and hyphal development than those carrying an OA moiety. In line with this observation, Rub W, which differs in the number of carbon atoms in the side chain, was less effective than Rub C. It is likely that more lipophilic compounds can penetrate biological membranes more easily [50]. As a result, lipophilic compounds may have better access to their target sites within the cell, leading to enhanced activity [51,52]. In the case of Rub C, the structure of the side chain resembles to some degree that of FA. Interestingly, Rub C demonstrated similar, or even stronger, activity than FA, which was used as the positive control. FA is a known inhibitor of hyphal growth and the biofilm formation of *C. albicans* [53,54,55]. 

As the treatment of different maturation stages of *C. auris* biofilms showed, Rub C prevented biofilm formation during the first stage of biofilm development with the highest efficacy when given shortly after the attachment phase. This efficacy was reduced when biofilms were treated after 12 h and 24 h of growth. Since the hyphae/pseudohyphae of *C. albicans* and *C. auris* are essential to attach to certain surfaces and to form robust biofilms [56,57], we hypothesized that Rub C might inhibit the formation of biofilms by affecting the growth of pseudohyphae or hyphae. Pseudohyphae in *C. auris* are known to adapt to stressful living conditions, such as high concentrations of salts [17,58]. In addition, the yeast-to-hyphae transition of *C. albicans* occurs during infections and allows for the pathogen to escape from macrophages by destroying the cell membrane via polarized growth [59]. This invasive growth can also cause damage to tissues by invading epithelial cells and causing bloodstream infections, resulting in larger damages in human hosts [60,61,62,63]. 

According to visualization via CLSM analysis, Rub C caused changes in the morphology of *C. auris* biofilms. Especially high concentrations lead to a very dense and compact surface-bound structure without visible hyphae, while a Rub C concentration of 62.5 µg/mL resulted in a more aggregated and porous-appearing biofilm structure. Furthermore, the results of CFU analysis revealed a significant increase in cell growth following 12 h of treatment with a concentration of 250 µg/mL of Rub C. However, after 24 h of incubation, the growth was inhibited compared to the control. This finding is consistent with the observations from CLSM images acquired at the same concentration of 250 µg/mL Rub C after 24 h of treatment, which also showed more biomass compared to the control; this indicates an increased growth along with reduced metabolic activity of the cells. This could be attributed to a strong, initial promotion of non-hyphae growth in the presence of 250 µg/mL Rub C, followed by a decrease in metabolically active cells due to nutrient limitations within the batch system after 24 h. This dense and thick cell layer at the surface also explains the increase in biomass in the CV assay of *C. auris* at high concentrations (>125 µg/mL).

Indeed, we could visualize that Rub C inhibited the pseudohyphae and hyphae formation of *C. auris* and *C. albicans* via light microscopy. Additionally, we performed a reporter gene assay which confirmed the inhibition of the hyphae development of *C. albicans* upon treatment with 12.5 µg/mL Rub C. The construct of the hyphal wall protein HWP1 promoter and the enzyme ß-galactosidase showed a reduction in enzymatic activity by 87% for Rub C and 39% for Rub W at concentrations above 12.5 µg/mL, while the other tested azaphilones only displayed inhibitory activity at the highest concentration (100 µg/mL). Based on these results, we assume that the biofilms of *C. auris* (treated with 0.5 µg/mL to 62.5 µg/mL Rub C) and *C. albicans* (treated with 7.8 µg/mL to 250 µg/mL Rub C) could be removed more easily by washing steps in the CV assays because they lacked pseudohyphae and hyphae—factors which are impeding the disturbance of *C. albicans* biofilms by external forces (e.g., vortexing, sonification) [56]. Similarly, biofilms containing pseudohyphae in *C. auris* possessed enhanced structural integrity compared to those lacking pseudohyphae [57].

EVs produced by *C. auris* are known to play crucial roles in various cellular processes [64]. Interestingly, in the SEM images of *C. auris*, we found that more and larger Evs were present after the treatment with high concentrations of Rub C (250 µg/mL) compared to the control. Thus, we not only observed growth-promoting effects on vegetative yeast cells along with inhibitory effects on pseudohyphae formation upon treatment with high concentrations of Rub C for *C. auris,* but also a release of EVs, in line with reports from *C. albicans* [65,66]. Due to shared biological processes in biofilm and hyphae regulation, *C. albicans* and *C. auris* might exhibit similar characteristics in EV production, as the study by Zamith-Miranda et al. let assume [64]. This multifactorial process might explain the differences in the structural architecture observed in the CLSM images. While high concentrations of Rub C promoted the growth of single yeast cells and resulted in a more compact and flattened biofilm structure, lower concentrations of Rub C did not stimulate vegetative growth, but still effectively inhibited the pseudo/hyphae formation of *C. albicans* and *C. auris*. 

## 5. Conclusions

Rub C, a fungal pigment derived from the stromata of *H. rubiginosum*, has demonstrated significant potential in inhibiting biofilm formation and yeast-to-hyphae transition against the two opportunistic fungal pathogens *C. albicans* and *C. auris*. This inhibition occurs in a concentration range below cytotoxic or lethal effects. Both biofilms and hyphae formation are critical virulence factors of these pathogens and likely strongly linked to each other. The encouraging aspect of our findings is that Rub C effectively targets these virulence factors without exhibiting cytotoxic effects on the tested mammalian cell lines, making it a promising candidate for therapeutic use in future. Potential applications could be the pre-therapeutical coating of medical devices; this is similar to the already investigated known biofilm inducer filastatin, which inhibited hyphal morphogenesis and the adhesion of *C. albicans* to polystyrene and human cells [67,68]. To gain a deeper understanding about the mechanisms of Rub C on both biofilm and hyphal inhibition, further investigations are needed to decipher the molecular mechanism. 

## Figures and Tables

**Table 1:** Antimicrobial activity of selected rubiginosin- and rutilin-type azaphilones against *C. albicans* and *C. auris*.

Tested Organisms	Strain No.	MIC [μg/mL]						
		Rub				Rut		Nystatin
		A	C	W	Z	A	B	
*C. albicans* [28]	DSM 1665	–	–	–	–	–	–	8.3
*C. albicans*	DSM 11225	–	–	–	–	–	–	8.3
*C. albicans* CAI-4 *HWP1-lacZ*	ZK3379	–	–	–	–	–	–	8.3
*C. auris*	DSM 21092	–	–	–	–	–	–	31.3

–: no activity.

**Table 2:** Cytotoxic activity of selected rubiginosin- and rutilin-type azaphilones against two cell lines.

Cell Lines		IC_50_ [μM]						
	Strain No.	Rub				Rut		Epothilone B
		A [28]	C	W	Z [28]	A	B	
KB-3-1	ACC 158	5.2	–	–	5.2	1.1	1.5	5.3 × 10^−5^/2.8 × 10^−5^ [27]
L929	ACC 2	3.2	–	–	4.7	1.2	2.2	1.7 × 10^−4^/3.1 × 10^−5^ [27]

–: no activity.

## Data Availability

All data generated are in the manuscript or the Supplementary Materials.

## Supplementary Materials

The following supporting information can be downloaded at: https://www.mdpi.com/article/10.3390/jof9070726/s1, Figure S1: Effects on biofilm formation of 2 h *C. auris* biofilms after different treatment time (8 h and 24 h) with Rub C. Methanol served as solvent control. Error bars indicate SD of duplicates in two biological repeats; *p* values: ** *p* < 0.01, *** *p* < 0.001. Figure S2: Volume of biomass for *C. auris* biofilms (24 h after exposure to Rub C of attached cells [2 h]) calculated by Imaris 9.31; Table S1: MIC assay experiment parameters. Table S2: Selected azaphilones were tested against the formation of biofilm of *S. aureus*, *P. aeruginosa* as well as *C. auris* and dispersal effect on preformed biofilm of *S. aureus* compared to solvent control (=0%), respectively. SDs are shown as ±SD. Table S3: Biofilm attachment of different developmental stages (2 h, 12 h, and 24 h old) of *C. auris* biofilm after treatment with positive control NYS compared to solvent control (=0%). SDs are shown as ±SD.
