# Supplementary material for: Inhibitory Effects of the Fungal Pigment Rubiginosin C on Hyphal and Biofilm Formation in Candida albicans and Candida auris
Source: J Fungi (Basel). 2023 Jul 5;9(7):726. doi: 10.3390/jof9070726 (PMC10381533; doi:10.3390/jof9070726)
Supplement: Supplementary file 1 [file jof-09-00726-s001.zip › jof-2477113-supplementary.pdf]

Inhibitory effects of the fungal pigment rubiginosin C on hyphal and biofilm formation in *Candida albicans* and *Candida auris*

Haoxuan Zeng, Marc Stadler, Wolf-Rainer Abraham, Mathias Müsken* and Hedda Schrey*

Isolation of selected azaphilones

Circa 2 g of stromata from *Hypoxylon rubiginosum* were used as starting material to isolate Rub C. Identification of the specimen was achieved by chemotaxonomic analysis [1]. The specimen was collected in June 2004 from unidentified dead wood in Parc Natural dels Aiguamolls de l'Empordà, Catalonia, Spain, by Barbara and Marc Stadler, and stored at the herbarium of the Helmholtz Centre for Infection Research (ID: STMA 04057).

The stromatal material was extracted by adding 500 mL acetone followed by ultrasonication at 40 °C for 1 h. This procedure was repeated once. Both acetone extracts were combined and dried in vacuum. This yielded ca.160 mg of crude extract.

In order to remove fatty acids and other lipophilic compounds, the crude extract was pre-fractionated using a Strata-X® C18-E cartridge (10 g/60 mL, SN: 8B-S001-HCH-T, Phenomenex, Torrance, CA/USA). The extract was dissolved in ca. 10 mL of acetone:acetonitrile (ACN) 1:1 and transferred onto the cartridge. Then, a two step-gradient of 20 mL acetone followed by 20 mL dichloromethane (DCM) was applied using a vacuum of ca. 800 mbar. The acetone fraction was dried in vacuo (yielding 148 mg) and used for further processing, as described below, while the DCM fraction was discarded.

The acetone fraction was dissolved in 10 mL (acetone : ACN : H_2O = 2:1:1) and separated using a PLC 2250 HPLC system (Gilson, Middleton, WI/USA) equipped with a Gemini C18 column (250×50 mm, 10 µm; Phenomenex) at a flow rate of 60 mL/min and the following gradient: 30% B (5 min), 30−45% (1 min), 45−55% (54 min), 55−100% (20 min), 100% (20 min). This led to pure 3 (10.7 mg, t_R: 84−84.5 min), whose structure was confirmed using spectral methods [1].

Other selected azaphilones were isolated and elucidated as procedure described previously [1]. 10 mg/ml stock solutions of compounds were prepared in 100% methanol. All compounds were fully dissolved in methanol.

Antimicrobial assay

The assay was conducted as a minimum inhibitory concentration (MIC) assay in 96-well round-bottom microtiter plates using the parameters summarized in table S1 and as already described in [2]. Stocks of the test organisms were generated by growing the organisms overnight in 250 mL (*Candida albicans* [DSM 11225], [DSM 1665], CAI-4 HWP1-*lacZ*, *Candida auris* [DSM 21092]) or 50 mL shaking (the rest organisms) flasks filled with 25 mL of the growth medium at 140 rpm (for media and temperatures see table S1). After incubation overnight, the OD of the suspension was measured and adjusted by diluting with the respective growth medium. OD_{600} nm was adjusted to 0.01 for bacteria and OD_{600} nm to 0.1 for fungi and 150 µL of the adjusted suspensions were added to all wells of a 96-well microtiter plate (one test organism per plate). Subsequently, additional 130 µL (1 mg/mL tested compounds) or 142.5 µL (10 mg/mL tested compounds) of suspensions, plus 20 µL (1 mg/mL tested compounds) respectively 7.5 µL (10 mg/mL tested compounds) of the test compounds were added to first row. Controls (one compound/column) were added as well: MeOH were used as solvent control, while different positive controls (reference drugs) were used for the according test organisms (see table S1). Starting from row A, 150 µL of the suspension were transferred

to the next row, the contents thoroughly mixed, and 150 µL transferred to the following row. The remaining 150 µL after row H were discarded. This resulted in a serial dilution of the test compounds, ranging from 66.7 µg/mL in row A to 0.52 µg/mL in row H (1 mg/mL tested compounds) or 250 µg/mL in row A to 2 µg/mL in row H (10 mg/mL tested compounds).
The 96-well microtiter plates were then incubated overnight on a microplate shaker at 800 rpm at 30 or 37 °C (see table S1) and were visually evaluated the next day. The MIC is defined as the lowest concentration where no growth of the test organism was observed. A lower MIC thus corresponds to a higher antimicrobial activity of the test compound.

Table S1: MIC assay experiment parameters.

Tested organisms	Strain No.	Growth medium	Incubation temp. [°C]	Positive control (reference)
C. albicans [1]	DSM 1665	RPMI 1640	37	nystatin 0.1 mg/mL
C. albicans	DSM 11225	RPMI 1640	37	nystatin 10 mg/mL
C. albicans CAI-4 *HWP1–lacZ*	Zk3379	RPMI 1640	37	nystatin 10 mg/mL
C. auris	DSM 21092	RPMI 1640	37	nystatin 10 mg/mL

Cytotoxicity assay
KB-3-1 (ACC 158) cervix carcinoma as well as L929 (ACC 2) mouse fibroblasts were cultured under standard cell culture conditions (37 °C, 5% CO_2, 95% humidified atmosphere). The cells were maintained in high glucose DMEM containing 10% fetal bovine serum and 100 U/mL penicillin and streptomycin (Gibco, Thermo Fisher Scientific, Waltham, USA). Cell lines were serially passaged after trypsinization, using 0.05% trypsin / 0.02% EDTA solution. The cultures were routinely monitored for potential contamination, and only mycoplasma free cultures were used. The test compounds were investigated for their anti-proliferative effect on cell lines, using the 3-(4,5-dimethylthiazol-2-yl)-2,5-diphenyltetrazolium bromide (MTT) based cell viability assay [3, 4]. Cells were seeded in 96 well microtiter plates (Sarstedt, Germany) with a cell density of 0.05 * 10^6 cells per mL and 100 µL per well and were incubated for 24 h. A dilution series of test compounds was added to the wells, ranging in eleven steps from 37 µg/mL – 0.63 ng/mL, and equal amounts of DMSO or methanol were added as solvent controls. Treated cells were incubated for a further 72 h. Then, 12.5 µL per well of MTT solution (0.05% in PBS) were added, followed by another 2 h of incubation. The plates were centrifuged, the medium was discarded and 25 µL per well of SDS solution in DMSO (10% SDS, 0.6% AcOH in DMSO) were added to dissolve the formazan. The plates were incubated for another hour. Absorbances at 570 nm and 630 nm were measured with a plate reader (Tecan, Männedorf, canton of Zürich, Switzerland). Background absorbance (630 nm) was subtracted from the formazan signal (570 nm). The resulting absorbance is directly proportional to the amount of viable cells. The control was normalized to 100% viable cells, and the viability of cells treated with the test compounds was calculated accordingly. IC_{50} values were calculated based on a sigmoidal fit model using GraphPad Prism. Means and SD were calculated from at least four independent experiments.

Biofilm inhibition assay
Selected azaphilones Rub C and W, Rut A and B were tested against the formation of biofilms in *Staphylococcus aureus* (DSM 1104). *S. aureus* DSM 1104 from a −20 °C stock was precultured in 25 mL CASO (casein-peptone soymeal-peptone) medium in a 250 mL flask at 37 °C with 100 rpm for 20 h. The OD_{600} of the culture solution was adjusted to 0.001 McFarland standard and incubated in 96-well microtiter plates (TPP tissue culture ref.no 92196, Switzerland) for 18 h at 37 °C with 150 µL of serially diluted test

compounds (250–2 µg/mL) in CASO with 4% glucose broth. The inhibition of biofilm formation was evaluated by staining with 150 µL of 0.1% crystal violet (CV; Thermo Fisher) following previously established protocols [5]. In the next step, the supernatant of the 96-well plate was discarded and the wells were rinsed once with PBS buffer. The remaining biofilms were stained at room temperature for 15 min, rinsed three times with PBS buffer, and finally dissolved in 150 µL ethanol (95%). The absorbance of the resulting solution at 530 nm was quantified using a plate reader (Synergy 2, BioTek). Methanol (2.5%) and microporenic acid A (MAA) [6] (250–2 µg/mL) were used as negative and positive controls. Standard deviations (SD) of two repeats with duplicates were 10% or less.

Selected azaphilones Rub C and W, Rut A and B were tested against the formation of biofilms in *Pseudomonas aeruginosa* (PA 14) DSM 19882. Bacterial strain was precultured in 25 mL LB medium (Luria-Bertani Broth) with a 250 mL flask at 37 °C with shaking 100 rpm overnight. The turbidity of the baterial solution was measured and diluted to match the turbidity of a 0.1 McFarland standard in M63 medium, which is supplemented with magnesium sulfate, glucose and casamino acids as previously described [5]. The compounds were added into 150 µL bacterial solution at the concentration (250–2 µg/mL) then the solution was added in U-bottom 96 well plate (Falcon ref.no 351177, Thermo Fisher Scientific). The plates were incubated at 37 °C at 150 rpm for 24 h and biofilms were established at the air liquid interface. The plates were rinsed once by using PBS buffer, the biofilms were stained by 150 µL 0.1% CV at room temperature for 15 min and then rinsed two times by using PBS buffer. The absorbance was quantified with the plate reader (Synergy 2, BioTek) at 550 nm using ethanol (95%). Methanol (2.5 %) and myxovalargin A (250–2 µg/mL) were used as negative control and positive control, respectively [6]. The assay was conducted for once.

Table S2: Selected azaphilones were tested against the formation of biofilm of *S. aureus*, *P. aeruginosa* as well as *C. auris* and dispersal effect on preformed biofilm of *S. aureus* compared to solvent control (= 0%), respectively. SDs are shown as ±SD.

Compounds	Strains	Concentrations [µg/mL]							
		250	125	62.5	31.3	15.6	7.8	3.9	2
**Rub A**	***C. auris***	/	/	/	/	/	/	/	/
**Rub C**	***S. aureus***	82 ±1	86 ±1	87 ±1	88 ±1	88 ±1	53 ±4	35 ±7	/
	**preformed *S.aureus***	/	/	/	/	/	/	/	/
	P. aeruginosa	/	/	/	/	/	/	/	/
**Rub W**	***S. aureus***	86 ±1	85 ±2	87 ±2	87 ±1	87 ±1	77 ±5	44 ±10	24 ±8
	**preformed *S.aureus***	/	/	/	/	/	/	/	/
	P. aeruginosa	/	/	/	/	/	/	/	/
**Rub Z**	***C. auris***	/	/	/	/	/	/	/	/
**Rut A**	***S. aureus***	60 ±6	55 ±9	51 ±10	51 ±10	21 ±2	/	/	/
	**preformed *S.aureus***	/	/	/	/	/	/	/	/
	P. aeruginosa	/	/	/	/	/	/	/	/
**Rut B**	***S. aureus***	84 ±2	85 ±2	86 ±2	86 ±2	87 ±2	88 ±1	88 ±1	86 ±2
	**preformed *S.aureus***	51 ±8	/	/	/	/	/	/	/
	P. aeruginosa	/	/	/	/	/	/	/	/
**MAA**	***S. aureus***	83 ±6	85 ±5	86 ±4	86 ±4	83 ±6	77 ±10	45 ±10	/
	**preformed *S.aureus***	65 ±4	65 ±7	60 ±9	54 ±6	33 ±10	/	/	/
**Myxovalargin A**	***P. aeruginosa***	55	65	59	61	/	/	/	/

NYS	*C. auris*	56 ±2	45 ±10	53 ±8	50 ±10	47 ±2	42 ±15	60 ±10	/
**(/) no activity**									

Table S3: Biofilm attachment of different developmental stages (2 h, 12 h, and 24 h old) of *C. auris* biofilm after treatment with positive control nystatin (NYS) compared to solvent control (=0%). SDs are shown as ±SD.

**Compound**	***C. auris* biofilm**	**Concentrations [µg/mL]**					
		**62.5**	**31.3**	**15.6**	**7.8**	**3.9**	**2**
	**2 h**	47 ±8	50 ±10	53 ±10	58 ±16	39 ±10	84 ±25
**NYS**	**12 h**	45 ±8	41 ±9	43 ±6	49 ±10	64 ±12	96 ±12
	**24 h**	46 ±5	50 ±10	49 ±5	51 ±9	48 ±2	93 ±28

Time dependent assay

The turbidity of *C. auris* (DSM 21092) dispersion was measured at 280 nm and diluted to the turbidity of a 0.5 McFarland standard. *C. auris* was cultured in RPMI 1640 medium supplemented with 0.165 mM MOPS (37 °C, 150 rpm, 2 h) in 96-well non-tissue microtiter plates (Falcon no. 351172, Thermo Fisher Scientific). After incubation, *C. auris* biofilms were washed once by PBS buffer and treated with serial diluted Rub C (250 – 0.02 µg/mL) in fresh RPMI 1640 medium supplemented with 0.165 mM MOPS for 8 h and 24 h at 37 °C with shaking at 150 rpm. Samples of each time point were further processed and evaluated by microtiter plate reader as described above. No significance was observed at 8 h for the sample treated with 15.6 µg/mL Rub C.

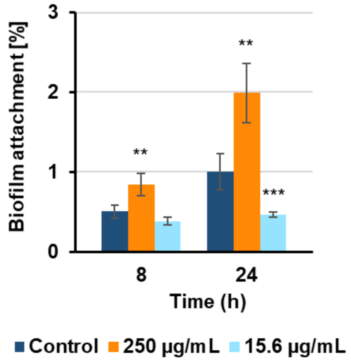

Figure S1. Effects on biofilm formation of 2 h *C. auris* biofilms after different treatment time (8 h and 24 h) with Rub C. Methanol served as solvent control. Error bars indicate SD of duplicates in two biological repeats; p values: ** $p < 0.01$, *** $p < 0.001$.

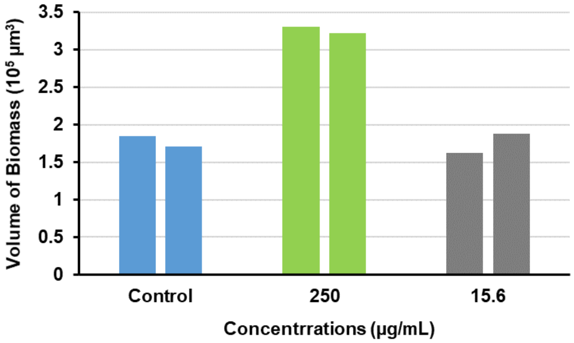

Figure S2. Volume of Biomass for *C. auris* biofilms (24 h after exposure to Rub C of attached cells [2 h]) calculated by Imaris 9.31.

References

1. Becker, K.; Kuhnert, E.; Cox, R. J; Surup, F. Azaphilone pigments from *Hypoxylon rubiginosum* and *H. texense*: absolute configuration, bioactivity, and biosynthesis. *Eur. J. Org. Chem.* **2021**, *36*, 5094–5103. doi: 10.1002/ejoc.202001661
2. Helaly, S. E.; Ashrafi, S.; Teponno, R. B.; Bernecker, S.; Dababat, A. A.; Maier, W.; Stadler, M. Nematicidal cyclic lipodepsipeptides and a xanthocillin derivative from a phaeosphariaceous fungus parasitizing eggs of the plant parasitic nematode *Heterodera filipjevi*. *J. Nat. Prod* **2018**, *81*, 2228–2234. doi: 10.1021/acs.jnatprod.8b00486
3. Becker, K.; Wessel, A.-C.; Luangsa-ard, J.J.; Stadler, M. Viridistratins A−C, Antimicrobial and cytotoxic benzo[*j*]fluoranthenes from stromata of *Annulohypoxylon viridistratum* (Hypoxylaceae, Ascomycota). *Biomol.* **2020**, *10*, 805. doi: 10.3390/biom10050805
4. Sandargo B.; Michehl, M.; Praditya, D.; Steinmann, E.; Stadler, M.; Surup, F. Antiviral meroterpenoid rhodatin and sesquiterpenoids rhodocoranes A–E from the wrinkled peach mushroom, *Rhodotus palmatus*. *Org. Lett*. **2019**, *21*, 3286. doi: 10.1021/acs.orglett.9b01017
5. O'Toole, G. A.; Kolter, R. Initiation of biofilm formation in *Pseudomonas* fluorescens WCS365 proceeds via multiple, convergent signalling pathways: a genetic analysis. *Mol. Microbiol.* **1998**, *28*, 449–461. doi: 10.1046/j.1365-2958.1998.00797.x
6. Gillsch, F.; Zeng, H.; Bär, S, I.; Schrey, H.; Schobert, R. Synthesis and bioactivity of ophiofuranones A and B. *J. Org. Chem.* **2022**, *87*, 6520–6523. doi: 10.1021/acs.joc.2c00521
